# Comparing Different Statistical Models and Multiple Testing Corrections for Association Mapping in Soybean and Maize

**DOI:** 10.3389/fpls.2019.01794

**Published:** 2020-02-25

**Authors:** Avjinder S. Kaler, Jason D. Gillman, Timothy Beissinger, Larry C. Purcell

**Affiliations:** ^1^ Department of Crop, Soil, and Environmental Sciences, University of Arkansas, Fayetteville, AR, United States; ^2^ Plant Genetic Research Unit, USDA-ARS, Columbia, MO, United States; ^3^ Division of Plant Breeding Methodology, Center for Integrated Breeding Research, Georg-August-Universität, Göttingen, Germany

**Keywords:** association mapping, genome-wide association analyses, multiple testing correction, statistical model analysis, quantitative trait loci

## Abstract

Association mapping (AM) is a powerful tool for fine mapping complex trait variation down to nucleotide sequences by exploiting historical recombination events. A major problem in AM is controlling false positives that can arise from population structure and family relatedness. False positives are often controlled by incorporating covariates for structure and kinship in mixed linear models (MLM). These MLM-based methods are single locus models and can introduce false negatives due to over fitting of the model. In this study, eight different statistical models, ranging from single-locus to multilocus, were compared for AM for three traits differing in heritability in two crop species: soybean (*Glycine max* L.) and maize (*Zea mays* L.). Soybean and maize were chosen, in part, due to their highly differentiated rate of linkage disequilibrium (LD) decay, which can influence false positive and false negative rates. The fixed and random model circulating probability unification (FarmCPU) performed better than other models based on an analysis of Q-Q plots and on the identification of the known number of quantitative trait loci (QTLs) in a simulated data set. These results indicate that the FarmCPU controls both false positives and false negatives. Six qualitative traits in soybean with known published genomic positions were also used to compare these models, and results indicated that the FarmCPU consistently identified a single highly significant SNP closest to these known published genes. Multiple comparison adjustments (Bonferroni, false discovery rate, and positive false discovery rate) were compared for these models using a simulated trait having 60% heritability and 20 QTLs. Multiple comparison adjustments were overly conservative for MLM, CMLM, ECMLM, and MLMM and did not find any significant markers; in contrast, ANOVA, GLM, and SUPER models found an excessive number of markers, far more than 20 QTLs. The FarmCPU model, using less conservative methods (false discovery rate, and positive false discovery rate) identified 10 QTLs, which was closer to the simulated number of QTLs than the number found by other models.

## Introduction

Connecting genotype to phenotype, known as genetic mapping, is important for modern crop breeding and improvement (Mackay, 2001). Two of the most commonly used approaches for genetic mapping are association mapping (AM) and biparental linkage mapping (LM). AM is an alternative approach to traditional mapping of biparental populations and is currently widely used in plant, animal (Goddard and Hayes, 2009), model species (Brachi et al., 2010), and human genetics (Risch and Merikangas, 1996; Nordborg and Tavaré, 2002). Most important traits in plants are complex and controlled by many genes and influenced by environment. With advancements in high throughput genotyping and sequencing technologies, single nucleotide polymorphisms (SNPs) provide relatively low cost and dense marker coverage across various genomes (Syvänen, 2005). Genotyping diverse lines provides thousands of SNPs across the genome that enables fine mapping complex trait variation down to nucleotide sequences by exploiting historical recombination events (Zhu et al., 2008). AM has lower overall statistical power to detect rare alleles and epistatic interactions than traditional LM, but it has several advantages, which include increased mapping resolution, broader allele coverage, reduced time and cost compared to developing biparental mapping populations, and potentially greater number of alleles evaluated (Yu et al., 2006).

Associating functional variants (alleles, loci) to phenotypes is a fundamental aim of both AM and LM (Botstein and Risch, 2003). The detection of quantitative trait loci (QTLs) through AM depends on the level of linkage disequilibrium (LD) between functional loci and markers. LD refers to a nonrandom association of alleles at different loci. LD is influenced by physical linkage and recombination, but it is a separate phenomenon—unlinked loci can be in a state of LD, and linked loci can be in a state of linkage equilibrium (Terwilliger and Weiss, 1998). The level of LD extent in a specific set of experimental genotypes can be measured statistically and has been widely leveraged in plants and animals to map and clone genes controlling complex genetic traits (Risch and Merikangas, 1996; Dunning et al., 2000; Pritchard and Przeworski, 2001; Nordborg and Tavaré, 2002). LD can be measured based upon the correlation between alleles at pairs of loci as physical distance between the loci increases. Outcrossing crop species, such as maize, have more genetic diversity (Remington et al., 2001; Yan et al., 2009) and also more rapid LD decay than self-pollinated species such as soybean, which has less overall genetic diversity (Gupta et al., 2006; Hyten et al., 2007; Schmutz et al., 2010; Song et al., 2015). Species with faster LD decay over physical distance, as compared to those with slow LD decay, require higher marker density over the genome to capture associations between marker and phenotype (Yu et al., 2006).

Several statistical models are available to identify associations between marker loci and numerous phenotypes that range from simple to increasingly complex. As genotypic data are becoming more readily available, accurately decoding the complexity of traits in a diverse population is only possible if accurate and more comprehensive statistical models can distinguish true biological associations from false positives arising from population structure and family relatedness without overcorrecting and resulting in false negatives. Using covariates for structure and kinship in the statistical model can control these confounding factors. STRUCTURE (Pritchard et al., 2000), principal component analysis (PCA) (Price et al., 2006), and a discriminant analysis of principal components (DAPC) (Jombart et al., 2010) are approaches that use genetic markers to determine population organization. Results from STRUCTURE and PCA are similar, but PCA is generally more commonly used due to lower required computational resources and time to generate covariates. False positives can also arise due to more recent common ancestry and family relatedness, which can be controlled by inclusion of a kinship matrix into the linear model. Identity-by-state is one of the most commonly used approaches to estimate familial relatedness among individuals in a diverse population (Loiselle et al., 1995).

The incorporation of population structure and a kinship matrix as covariates in mixed linear models (MLM) has become a popular approach to control false positives. Since the first MLM of AM was published by Yu et al. (2006), many MLM-based methods have been proposed (Zhang et al., 2010; Wang et al., 2014). All these models are single-locus models, which means that they comprise a one-dimensional genome scan by testing one marker at a time, iteratively for every marker in a dataset. This single-locus approach fails to match the true genetic model of complex traits that are controlled by numerous loci simultaneously. To cope with this problem, multilocus AM models have been recommended because these models consider the information of all loci simultaneously (Wang et al., 2016). MLM-based models can also induce false negatives due to over fitting of the model where some potentially important associations can be missed (Liu et al., 2016).

False negatives in AM can result when multiple comparison adjustments are used to determine statistical significance. Two commonly used multiple comparison methods in AM are Bonferroni correction (Holm, 1979) and false discovery rate (FDR) (Benjamini and Hochberg, 1995), which select the significant threshold. However, overly conservative thresholds can lead to high false negative error rates. Therefore, selection of an appropriate model and threshold are important steps in identifying markers that are truly associated with specific traits and which could be located within or very close to genes that control the trait variation, while controlling both false-positive and false-negative associations.

The objective of this study was to compare eight different AM statistical models, ranging from single to multilocus, for three previously reported traits and six simulated traits in soybean and maize. These crops were selected because of their difference in LD as indicated by the LD decay rate: maize, which is naturally outcrossing, displays much more rapid LD decay than soybean, a self-pollinating species. We also compared these eight statistical models for six qualitative traits in soybean, all of which have known causal genes with published genomic positions. Finally, we evaluated five multiple comparison methods when used in conjunction with these eight AM models.

## Materials and Methods

### Data Collection

This study included three datasets collected from previously published or online sources (referred to as “previously reported traits” subsequently). These previously reported datasets were the best linear unbiased predictions (BLUP) values across different environments for each trait. We also simulated six datasets from two crop species: soybean and maize. Previously reported data for soybean included canopy wilting (CW) with a broad sense heritability (H) of 80% (Kaler et al., 2017a), carbon isotope ratio (δ^13^C, H = 60%, Kaler et al., 2017b), and oxygen isotope ratio (δ^18^O, H = 20%, Kaler et al., 2017b). For maize, the previously reported data included days to tasseling (DT, H = 85%), ear height (EH, H = 80%), and ear diameter (ED, H = 85%) (Flint-Garcia et al., 2005). For both soybean and maize, six traits were simulated that varied in heritability and the number of QTLs (Q). These simulated traits were generated using the same genotypic markers that were used for AM of previously reported data. These six simulated datasets for each crop had varying heritabilities and genetic architectures. We simulated traits with H = 20% and Q = 20 (H20_Q20), H = 60% and Q = 20 (H60_Q20), H = 80% and Q = 20 (H80_Q20), H = 20% and Q = 40 (H20_Q40), H = 60% and Q = 40 (H60_Q40), and H = 80% and Q = 40 (H80_Q40). The R-script to generate the simulated data sets is provided in a supplement (**Table S1**). These data were simulated to have random QTLs effects. The simulated data for soybean and maize are provided in **Supplementary Data Files 1** and **2**, respectively. Previously reported data of soybean consisted of 346 accessions as described by Kaler et al. (2017b), and previously reported data of maize (Flint-Garcia et al., 2005) consisted of 279 accessions from the Panzea database website (www.panzea.org).

### Genotypic Data and LD

Genotypic data for both crops consisted of SNP markers. In soybean, SNP data were obtained from the Illumina Infinium SoySNP50K iSelect SNP BeadChip that provided 42,509 SNP markers for all 346 accessions (Song et al., 2013; Song et al., 2015). In maize, SNP data were obtained from the Illumina MaizeSNP50 BeadChip that provided 50,896 SNP markers for 273 accessions (Flint-Garcia et al., 2005). Quality control checks were performed, which included removing monomorphic markers, markers with minor allele frequency (MAF) ≤ 5%, and markers with a missing rate higher than 10%. An LD-kNNi method, which is based on a k-nearest-neighbor-genotype, was applied to impute the remaining missing marker datasets (Money et al., 2015).

After performing quality controls, 31,260 SNPs for soybean and 48,833 SNPs for maize with MAF > 5% were used for AM. For maize, SNPs were more or less equally distributed across the genome for both euchromatic and heterochromatic regions (**Figures S1**). For soybean, SNPs were not equally distributed across the genome; there was higher marker density in euchromatic than heterochromatic regions (**Figure S2**). All chromosomes of maize had more SNPs than those of soybean (**Table S2**). The decay rate of LD was estimated using the GAPIT R package (Lipka et al., 2012). The decay rate of LD was much greater in maize than soybean with an average LD across all chromosomes decaying to *r* ^2^ = 0.25 in less than 1 kb. In comparison, in soybean, an average LD across all chromosomes decayed to *r* ^2^ = 0.25 in approximately 2,000 kb (**Figure S3**). In soybean, LD decay rates were different in euchromatic and heterochromatic regions (Hyten et al., 2007; Schmutz et al., 2010; Kaler et al., 2017a; Kaler et al., 2017b). Using both regions together affected the results of LD decay rate.

Broad sense heritability of traits was calculated using the formula: $H^{}=\;\sigma_{G}^{2}\;/\;\left(\sigma_{G}^{2}+\left(\frac{\sigma_{\epsilon }^{2}}{r}\right)\right)$, where $\sigma_{G}^{2}$ is the genotypic variance, $\sigma_{\epsilon }^{2}$ is the residual variance, and *r* is the number of replications. Marker-based narrow sense heritability (*h^2^*) was estimated to understand the variation and trend of predictive ability across traits (Kruijer et al., 2015) using the GAPIT R package. In the GAPIT package, the MLM model can be described as: *Y*=*Xβ*+*Zu*+*e*, where Y is the vector of observed phenotypes; β is an unknown vector containing fixed effects, including the genetic marker, population structure (Q), and the intercept; u is an unknown vector of random additive genetic effects from multiple background QTL for individuals/lines; X and Z are the known design matrices; and e is the unobserved vector of residuals. The u and e vectors are assumed to be normally distributed with a null mean and a variance of: $Var\;\left(\begin{array}{c} u\\ e \end{array}\right)=\left(\begin{array}{cc} G & 0\\ 0 & R \end{array}\right)$, where G = σ^2^_a_K with σ^2^_a_ as the additive genetic variance and K as the kinship matrix. Homogeneous variance is assumed for the residual effect; i.e., R = σ^2^_e_I, where σ^2^_e_ is the residual variance. The proportion of the total variance explained by the genetic variance is defined as marker-based heritability.

### Description of AM Models

The eight AM models evaluated ranged from simple to complex and included: (i), analysis of variance (ANOVA), (ii) general linear model (GLM) with PCA (principle component analysis) (Price et al., 2006), (iii) MLM with PCA + K (Kinship matrix for family relatedness estimates) (Yu et al., 2006), (iv) compressed MLM (Zhang et al., 2010), (v) enriched compressed MLM (Li et al., 2014), (vi) settlement of MLM under progressively exclusive relationship (SUPER) (Wang et al., 2014), (vii) multiple loci MLM (MLMM) (Segura et al., 2012), and (viii) fixed and random model circulating probability unification (FarmCPU) (Liu et al., 2016). Models from (i) to (vi) are single locus models, and (vii) and (viii) are multilocus models. **Table 1** lists and briefly summarizes keys aspects of models evaluated in the present study.

**Table 1:** Description of eight genome-wide association mapping models.

Model	Description	References
Analysis of variance (ANOVA)	Single locus analysis, the null hypothesis of an ANOVA using a single SNP is that there is no difference between the trait means of any genotype group.	Lewis, 2002
General Linear Model (GLM)	Single locus analysis, the GLM uses principle components as covariates in the model to reduce the false positives that arise due to only population structure.	Price et al., 2006
Mixed Linear Model (MLM)	Single locus analysis, the MLM uses principle components and kinship matrix in the model to reduce the false positives that arise from the family relatedness and population structure.	Yu et al., 2006
Compressed MLM (CMLM)	Single locus analysis, the CMLM clusters the individuals into groups and fits genetic values of groups as random effects in the model that improves statistical power compared to regular MLM methods.	Zhang et al., 2010
Enriched CMLM (ECMLM)	Single locus analysis, the ECMLM calculates kinship using several different algorithms and then chooses the best combination between kinship algorithms and grouping algorithms.	Li et al., 2014
Settlement of MLM Under Progressively Exclusive Relationship (SUPER)	Single locus analysis, the SUPER model uses the associated genetic markers (pseudo Quantitative Trait Nucleotides), instead of all the markers, to derive kinship. Whenever a pseudo QTN is correlated with the testing marker, it is excluded from those used to derive kinship.	Wang et al., 2014
Multiple Loci Mixed Linear Model (MLMM)	Multi-locus analysis, the MLMM incorporates a kinship matrix and selected cofactors, performed better with regard to the false-discovery rate and the QTL detection power than a model incorporating only a kinship matrix or only cofactors.	Segura et al., 2012
Fixed and random model Circulating Probability Unification (FarmCPU)	Multi-locus analysis, this model uses a modified MLM method, Multiple Loci Linear Mixed Model (MLMM), and incorporates multiple markers simultaneously as covariates in a stepwise MLM to partially remove the confounding between testing markers and kinship. To completely eliminate the confounding, MLMM is divided into two parts: Fixed Effect Model (FEM) and a Random Effect Model (REM) and uses them iteratively. FEM contains testing markers, one at a time, and multiple associated markers as covariates to control false positives. To avoid model over-fitting in FEM, the associated markers are estimated in REM by using them to define kinship. The *P*-values of testing markers and the associated markers are unified at each iteration.	Liu et al., 2016

The GLM with PCA model is expected to reduce the false positives that arise due to only population structure (Price et al., 2006). The MLM with PCA and K model includes the kinship matrix in the model and is expected to reduce the false positives that arise from family relatedness (Yu et al., 2006). Both GLM and MLM are reported to control false positives better than ANOVA (Price et al., 2006; Yu et al., 2006). The MLM model is reported to perform better than the GLM model alone by controlling false positives (Yu et al., 2006). Advantages of the MLM model to control false positives disappear for complex traits when they are associated with population structure having extensive genetic divergence. The MLM model controls the *P*-value inflation well, but it also leads to false negatives, thereby weakening identification of true associations (Zhang et al., 2010). To deal with this problem, the compressed MLM model (CMLM) was developed, which clusters the individuals into groups and fits genetic values of groups as random effects in the model (Zhang et al., 2010). The CMLM method improves statistical power compared to regular MLM methods (Zhang et al., 2010). Another suggested way to deal with *P*-value deflation due to MLM is to use a SUPER model in which only the associated genetic markers, instead of all the markers, are used as pseudo Quantitative Trait Nucleotides (QTNs) to derive kinship (Wang et al., 2014). Whenever a pseudo QTN is correlated with the testing marker, it is excluded from those used to derive kinship. The SUPER model applies a threshold on LD between the pseudo QTNs and the testing marker. This method improves the statistical power compared to using overall kinship from all markers.

FarmCPU is a multilocus model that was developed to control false positives without comprising false negatives (Liu et al., 2016). This model is not used extensively for AM of complex traits in crops because it has not been compared with other models for previously reported and simulated data. The FarmCPU model uses a modified MLM method, multiple loci linear mixed model (MLMM), and incorporates multiple markers simultaneously as covariates in a stepwise MLM to partially remove the confounding between testing markers and kinship. To completely eliminate the confounding, MLMM is divided into two parts: fixed effect model (FEM) and a random effect model (REM) and uses them iteratively. FEM contains testing markers, one at a time, and multiple associated markers as covariates to control false positives. To avoid model overfitting in FEM, the associated markers are estimated in REM by using them to define kinship. The *P*-values of testing markers and the associated markers are unified at each iteration. This model reportedly improves statistical power, increases computational efficiency, and the ability to control false positives and false negatives as compared to other models (Liu et al., 2016).

### Interpretation of Q-Q Plots and Model Evaluation

Examining quantile-quantile (Q-Q) plots is one of the most common ways of determining if models control false positives and false negatives (Stich et al., 2008; Stich and Melchinger, 2009; Würschum et al., 2012; Riedelsheimer et al., 2012; Kristensen et al., 2018). The Q-Q plot shows the expected negative-log of association probability (X-axis) across all markers versus the observed negative-log of association probability values (Y-axis). If a Q-Q plot has a straight line close to the 1:1 line without any tail, then it follows a uniform distribution, which means the null hypothesis is true and that there is no significant association or causal polymorphism. Any deviation of this straight line would indicate that the null hypothesis was not true and there were significant associations present. If the Q-Q plot does not have a straight line and tail, it indicates that there are false positives when a line inflates upward and there are false negatives when line deflates downward. If a Q-Q plot has a straight line, close to the 1:1 line, with a sharp upward deviated tail, it indicates that both false positives and false negatives were controlled, and that there are true associations and causal polymorphisms. This happens because most of the *P*-values observed follow a uniform distribution (i.e., they are not in LD with a causal polymorphism, so the null hypothesis is true) but the few that are in LD with a causal polymorphism will produce significant *P*-values [extremely low = extremely high -log (*P*-values)] and these are in the “tail”.

We evaluated these eight models for false positives and false negatives based on the Q-Q plots. A sharp deviation from the expected *P*-value distribution in the tail area would indicate that a model appropriately controlled both false positives and false negatives. Models were also compared using qualitative traits in soybean, which have known published genes for flower color (Takahashi et al., 2010), stem termination (Bernard, 1972), seed-coat luster (Gijzen et al., 2003), seed-coat color (Clough et al., 2004), hilum color (Carpentieri-Pipolo et al., 2015), and pubescence color (Toda et al., 2002; Zabala and Vodkin, 2003). Models were also compared using simulated data in which there were a known number of QTLs in the simulated data. The accuracy of a model was evaluated by identifying the number of QTLs in the simulated data.

### Evaluation of Multiple Comparisons Methods for AM

Three common multiple comparison methods were compared for determining statistical significance with a cutoff of *P =* 0.05. These methods included Bonferroni, false discovery rate, and positive false discovery rate. These comparisons were made using the PROC MULTTEST procedure of SAS version 9.4 (SAS Institute, 2013). The models were also compared to no multiple comparison adjustment at a *P*-value of 0.0003.

## Results

### Phenotype Descriptions

There were broad phenotypic ranges for all the traits evaluated in both soybean and maize (**Table 2**), which is required for dissecting complex traits through association analysis (McCarthy et al., 2008). Among the three traits in maize, broad and marker-based narrow sense heritability ranged between 80% to 85% and 70% to 80%, respectively. Among the three traits in soybean, broad and marker-based narrow sense heritability ranged between 20% to 80% and 3% to 71%, respectively (**Table 2**).

**Table 2:** Descriptive statistics of days to tasseling (DT), ear height (EH), and ear diameter (ED) in maize, and canopy wilting (CW), carbon isotope ratio (*δ* ^13^C), and oxygen isotope ratio (*δ* ^18^O) in soybean.

	Maize			Soybean		
	DT	EH	ED	CW	*δ* ^13^C	*δ* ^18^O
Mean	67.58	61.38	36.74	16.99	−29.06	20.87
Standard Deviation	5.75	20.27	4.05	6.46	0.27	0.43
Minimum	54.50	8	23.72	7.50	−29.81	19.20
Maximum	85.00	136	46.35	45.63	−28.37	22.29
Skewness	0.41	0.64	−0.29	1.39	−0.12	−0.11
Range	30.50	128.00	22.63	38.13	1.46	3.09
Count	279	279	279	346	346	346
Broad sense heritability (%)	85	80	85	80	60	20
Narrow sense heritability (%)	70	72	80	71	29	3

### Model Comparison With Soybean Data

**Figure 1:**
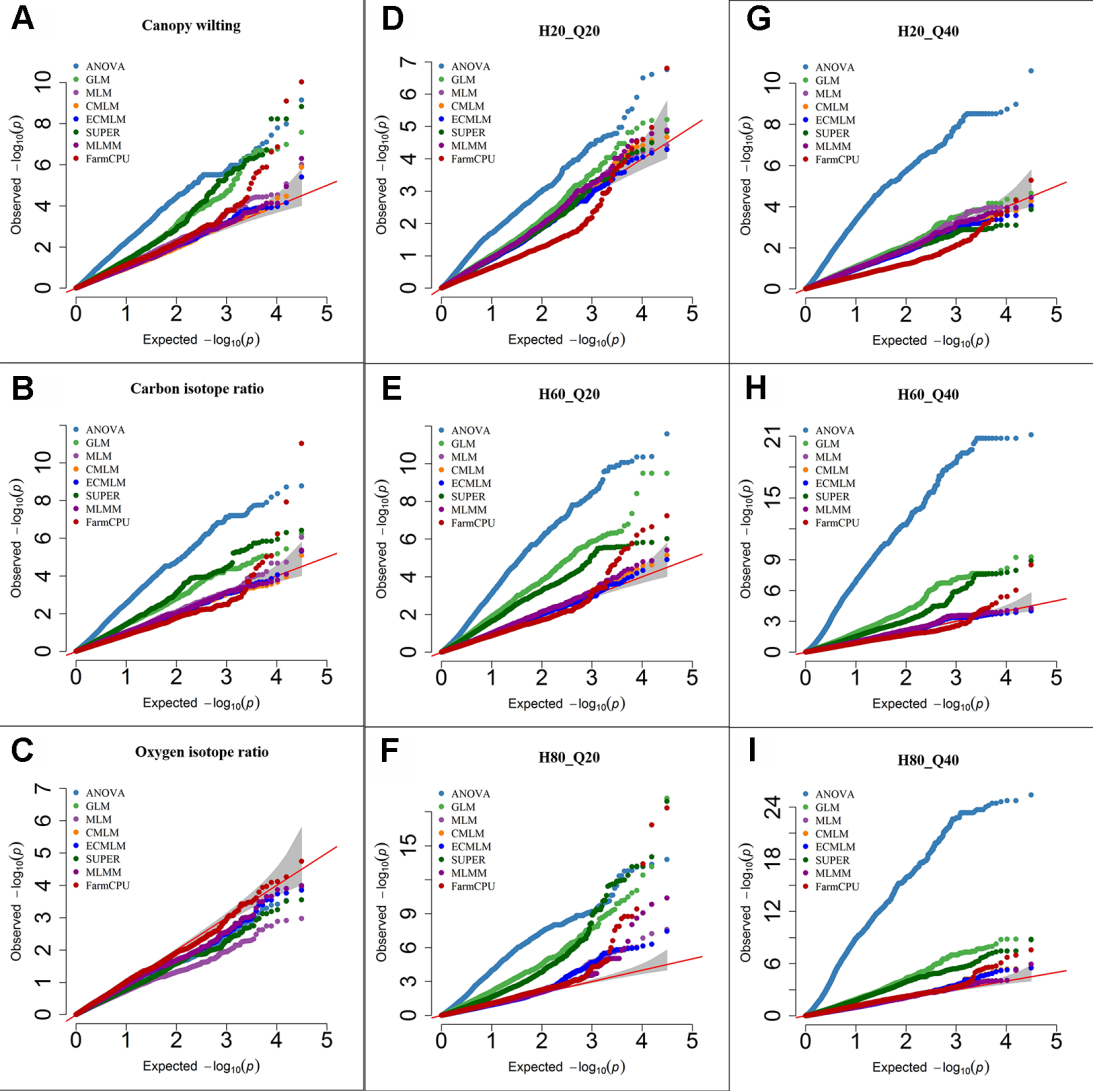
Quantile-quantile (QQ) plots of the eight models including Analysis of Variance (ANOVA), General Linear Model (GLM), Mixed Linear Model (MLM), Compressed MLM (CMLM), Enriched Compressed MLM (ECMLM), Settlement of MLM Under Progressively Exclusive Relationship (SUPER), Multiple Loci Mixed linear Model (MLMM), and Fixed and random model Circulating Probability Unification (FarmCPU) for three real traits including canopy wilting **(A)**, carbon isotope ratio **(B)**, and oxygen isotope ratio **(C)**, and six simulated traits that varied in heritability (H) and quantitative trait loci (Q) including H = 20% and Q = 20 (H20_Q20) **(D)**, H = 60% and Q = 20 (H60_Q20) **(E)**, H = 80% and Q = 20 (H80_Q20) **(F)**, H = 20% and Q = 40 (H20_Q40) **(G)**, H = 60% and Q = 40 (H60_Q40) **(H)**, and H = 80% and Q = 40 (H80_Q40) **(I)** in Soybean. The grey area represents the 95% concentration band.

**Figure 2:**
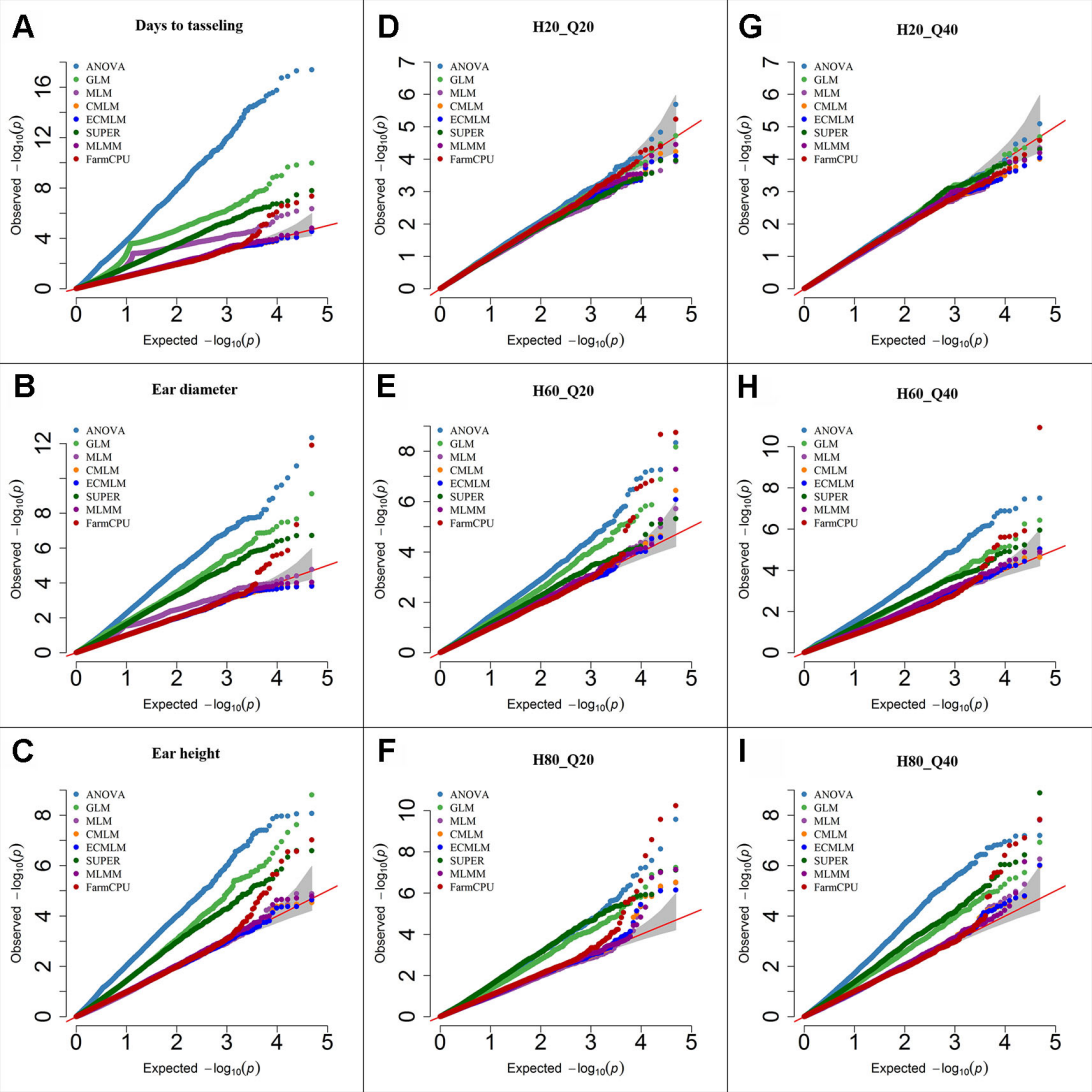
Quantile-quantile (QQ) plots of the eight models including Analysis of Variance (ANOVA), General Linear Model (GLM), Mixed Linear Model (MLM), Compressed MLM (CMLM), Enriched Compressed MLM (ECMLM), Settlement of MLM Under Progressively Exclusive Relationship (SUPER), Multiple Loci Mixed linear Model (MLMM), and Fixed and random model Circulating Probability Unification (FarmCPU) for three real traits including days to tasseling **(A)**, ear diameter **(B)**, and ear height **(C)**, and six simulated traits that varied in heritability (H) and quantitative trait loci (Q) including H = 20% and Q = 20 (H20_Q20) **(D)**, H = 60% and Q = 20 (H60_Q20) **(E)**, H = 80% and Q = 20 (H80_Q20) **(F)**, 2 = 20% and Q = 40 (H20_Q40) **(G)**, H = 60% and Q = 40 (H60_Q40) **(H)**, and H = 80% and Q = 40 (H80_Q40) **(I)** in Maize. The grey area represents the 95% concentration band.

Eight different AM models that ranged from simple to complex were compared using three previously reported traits and six simulated traits for soybean and maize (**Figures 1** and **2**). These eight AM models identified different numbers of significant markers associated with the previously reported and simulated traits for soybean when we consider the same significance threshold (**Table 3**). For example, if we consider the significance threshold as −Log10 (P) > 3.5 to declare a significant association for a simulated trait with 20 QTLs, we identified 2465 SNPs from ANOVA, 520 from GLM, 24 from MLM, 24 from CMLM, 16 from ECMLM, 229 from SUPER, 26 from MLMM, and 19 from FarmCPU (**Table 3**). All models, except the FarmCPU and MLMM, identified multiple significant SNP marker associations in close physical distance on the chromosome. These large peaks were generated because one SNP from these peaks had the highest significant association with traits, but the other markers at a given peak were in high LD with this most significant marker.

**Table 3:** Comparison of the number of significant markers (*P* ≤ 0.05) identified by multiple comparison methods including Bonferroni (Bon), false discovery rate (FDR), and positive false discovery rate (PFDR) using a simulated trait for soybean that had a heritability 60% and 20 QTLs (H60_Q20) in eight different association models eight including analysis of variance (ANOVA), general linear model (GLM), mixed linear model (MLM), compressed MLM (CMLM), enriched compressed MLM (ECMLM), settlement of MLM under progressively exclusive relationship (SUPER), multiple loci mixed linear model (MLMM), and fixed and random model circulating probability unification (FarmCPU).

	−Log_10_ *P* ≥ 3.5	Bon	FDR	PFDR
**ANOVA**	2,465	411	7,204	9,760
**GLM**	520	38	1,336	1,966
**MLM**	24	0	0	0
**CMLM**	24	0	0	0
**ECMLM**	16	0	0	0
**SUPER**	229	5	327	521
**MLMM**	26	0	0	0
**FarmCPU**	19	4	10	10

For comparative purposes, a *P*-value threshold (–Log_10_ *P* ≥ 3.5) without any adjustment is included.

For the CW trait (H = 80%), the ANOVA, GLM, and SUPER models had a large number of false positives as indicated by a substantial inflation of *P*-values (**Figure 1A**). Q-Q plots of complex models including MLM, CMLM, and ECMLM had a straight line with a slightly deviated tail, which indicated that these models reduced the false positives. However, most markers were close to the straight line of 1:1, indicating that they may have been reported as false negatives (**Figure 1A**). In contrast, the FarmCPU model followed a straight line close to 1:1, with a sharp upward deviated tail, indicating that this model controlled both false positives and false negatives (**Figure 1A**). For δ^13^C (moderate H = 60%), results of all models were similar to the CW trait, indicating that the FarmCPU model controlled both false positives and false negatives more effectively than other models (**Figure 1B**). For a low heritability trait, δ^18^O, the Q-Q plot for all models, except FarmCPU, deflated downward, indicating that these models increased false negatives. In contrast, Q-Q plots of the FarmCPU model for δ^18^O had a straight line close to the 1:1 with a slightly deviated tail, indicating that FarmCPU controlled both false positives and false negatives (**Figure 1C**).

Results from Q-Q plots of the six simulated traits in soybean were consistent with results from the previously reported data (**Figures 1D–I**). That is, ANOVA, GLM, and SUPER models had an inflation of *P*-values indicating there were a large number of false positives whereas MLM, CMLM, ECMLM, and MLMM controlled false positives but not false negatives. The Q-Q plots for FarmCPU indicated control of both false negatives and false positives. For all simulated traits, the ANOVA model had a large number of false positives because it inflated the *P*-value in the Q-Q plots (**Figures 1D–I**). When a simulated trait had a low heritability (H = 20) and a large QTL number (40), all complex models that incorporated the PCs and kinship matrix increased the number of false negatives, except the FarmCPU model (**Figure 1G**). When a simulated trait had a high heritability with 20 or 40 QTLs, complex models that included both PCs and kinship matrix reduced the false positives (**Figures 1F, I**), but still the FarmCPU model had a straight line that followed the 1:1 line with a sharp deviated tail compared to other models.

### Maize

In maize, we observed large effects of population structure and family relatedness. Similar to soybean (**Table 3**), the models that included the PCs and kinship matrix for maize identified a smaller number of markers than models that did not (data not shown). Likewise, the models that had no adjustment (ANOVA) or included only PCs (GLM) increased the number of significant markers for both previously reported and simulated traits when a specific threshold level was used compared with other complex models (**Table 3**). All single-locus models gave a peak of multiple, significant SNPs, which may result in missing the identification of other important genomic regions that may not have that high level of significance (*P*-value) as the markers in the peak region that are in high LD with the most significant marker. However, the multilocus model, FarmCPU and MLMM, did not show any clusters of significant markers in maize; instead they provided the highest significant marker at a specific genomic location, which led to identification of more markers at different locations (data not shown). Based on the Q-Q plots for all previously reported and simulated traits, the FarmCPU model performed much better than other models as indicated by the Q-Q plots with a straight line close to the 1:1 line with most sharply deviated tail (**Figure 2**).

### Qualitative Traits of Soybean

**Figure 3:**
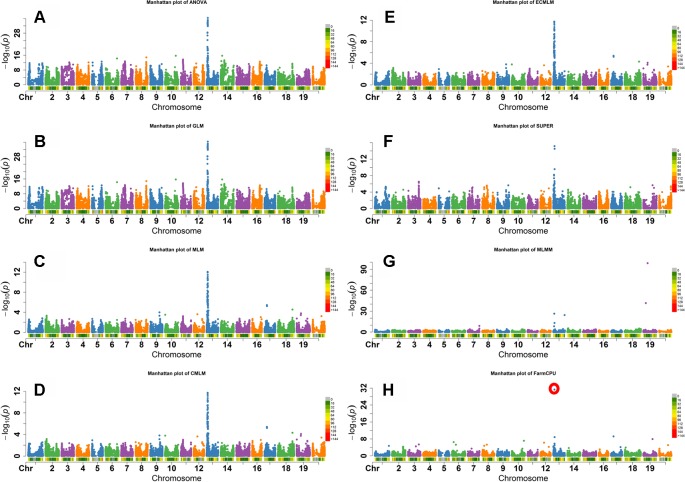
Manhattan plots of -Log10 (P) vs. chromosomal position of SNP markers associated with flower color in soybean from eight models including Analysis of Variance (ANOVA) **(A)**, General Linear Model (GLM) **(B)**, Mixed Linear Model (MLM) **(C)**, Compressed MLM (CMLM) **(D)**, Enriched Compressed MLM (ECMLM) **(E)**, Settlement of MLM Under Progressively Exclusive Relationship (SUPER) **(F)**, Multiple Loci Mixed linear Model (MLMM) **(G)**, and Fixed and random model Circulating Probability Unification (FarmCPU) **(H)**.

Flower color in soybean is a qualitative trait that is conferred by the *W1* gene. A small (65 bp) insertion of tandem repeats in exon 3 that truncates the translation product prematurely, resulting in a white flower instead of the wild-type purple flower (Zabala and Vodkin, 2005; Zabala and Vodkin, 2007). The *W1* locus is located on Gm13 at 4552540-4557331 base pairs in the Wm82.a1.v1.1 genomic assembly (Schmutz et al., 2010). As both alleles are widespread in soybean germplasm, this trait is ideal to determine which model would best identify markers closely linked to the known causative allele. The FarmCPU, GLM, and ANOVA models identified the most significant SNP associated with flower color on Gm13 (**Figure 3**). Other models, except the MLMM, identified most significant markers at different positions on Gm13 that were further away from the published gene on the same chromosome. For example, MLM identified the highest significant SNP at 3,822,639 base pairs. MLMM identified the highest significant SNP on Gm19. Unlike the other models, FarmCPU identified only the single SNP on Gm13 at the position 4,559,799 bp, closest to the position of *W1*gene, in the Wm82.a1.v1.1 genomic assembly (Schmutz et al., 2010) (**Figure 3**).

**Figure 4:**
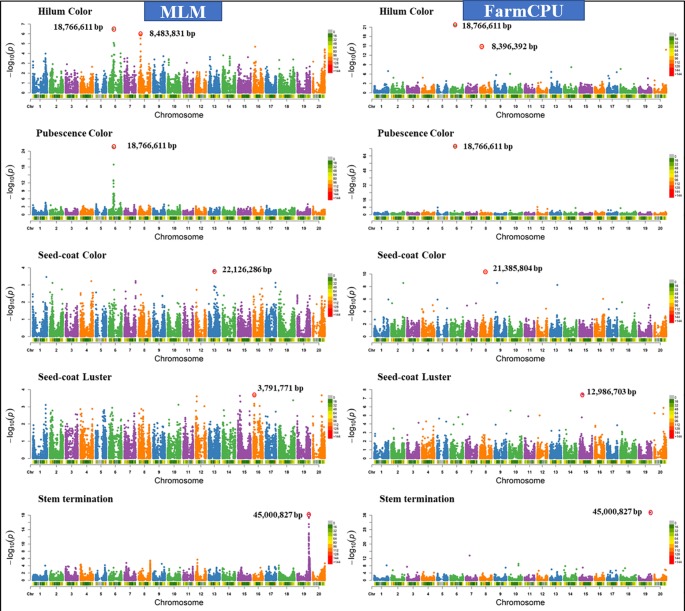
Manhattan plots of −Log10 (P) vs. chromosomal position of SNP markers associated with five qualitative traits in soybean from two models including, Mixed Linear Model (MLM) and Fixed and random model Circulating Probability Unification (FarmCPU).

Similar results were observed when models were compared using five other qualitative traits in soybean including hilum color, pubescence color, seed-coat color, seed-coat luster, and stem termination (determinacy) (data not shown). **Figure 4** shows the comparison of FarmCPU models with MLM for different qualitative traits. We chose a comparison of FarmCPU with MLM because it is a commonly used model for AM. The FarmCPU model identified a single significant SNP close to the genes associated with qualitative traits, instead of identifying a large peak of SNPs with MLM (**Figure 4**). For example, the FarmCPU model identified the single most significant SNP associated with stem termination on Gm19 at the position 45,000,827 base pairs closest to the position of the *Dt1* gene (45,183,357– 45,185,175 base pairs), instead of a large peak of SNPs with MLM. For three qualitative traits including, hilum color, pubescence color, and stem termination, the identified significant SNPs with the largest –Log_10_ *P*-value from the peak were similar to the position of the peak identified by the FarmCPU model (**Figure 4**). The most significant marker for hilum color and pubescence color was on Gm06 at the position 18,766,611 base pairs which was 28,586 base pairs distant from the T locus. Pubescence coloration and hilum coloration are in part determined by loss of function mutation affecting the Glyma06g21920 gene which results in grey pubescence at plant maturity and, in the right genetic background, can result in buff or imperfect hila (Zabala and Vodkin, 2003).

For seed-coat color and luster, the MLM identified the most significant SNPs on different chromosomes compared to the FarmCPU model (**Figure 4**). For seed-coat color, the FarmCPU model identified the most significant SNP on Gm8 at 21,385,804 base pairs close to the *Glyma08g27050* gene (21392963-21395430 bp), which is involved in flavonol biosynthesis (Palmer et al., 2004; Zabala and Vodkin, 2007; Yang et al., 2010). This marker association is most likely reporting the natural gene silencing cluster which results in yellow seed-coats (Clough et al., 2004; Tuteja et al., 2004) However, MLM identified the most significant SNP on Gm13 at the position 22,126,286 base pairs, where there was no gene present associated with seed coat color. For seed-coat luster, the FarmCPU model identified the most significant SNP on Gm15 at 12,986,703 base pairs within a gene *Glyma15g16670* (12,982,823-12,987,622), which is involved in a function of epidermis development. In contrast, MLM identified the SNP on Gm16 at 3,791,771 base pairs, which was not located close to any known gene for seed coat luster. Seed coat color and luster are controlled by more than one gene, hence, the FarmCPU model identified additional significant SNPs on other chromosomes, and all those regions are located close to previously reported genes for these traits (Palmer et al., 2004; Yang et al., 2010).

### Multiple Comparisons Methods for AM

Different multiple comparison methods were compared for determining statistical significance with a cutoff of *P* = 0.05. These comparison methods included: Bonferroni, false discovery rate, and positive false discovery rate (Holm, 1979; Hommel, 1988; Hochberg, 1988; Benjamini and Hochberg, 1995). The significance level of –Log10 (*P > *1.3, which is an equivalent to the *P* < 0.05) was used as a threshold before and after performing multiple comparison adjustments. We compared these methods for all traits in maize and soybean, but for brevity, we only show results of the simulated trait, H60_Q20 in soybean (**Table 3**). Results of all other traits were consistent with this trait (data not shown). These results indicated that these multiple comparison methods for AM were very conservative and only depended on the *P*-value of the association test. These results were evaluated based on the number of markers identified after adjustments. If the number of markers identified was more than 20, it indicates that there were false positives. If the number of markers identified was less than 20, it indicates that there were false negatives. In this study, ANOVA, GLM, and SUPER models had very large –Log10 *P*-values for significant associations; when multiple comparisons adjustments were performed, all 20 QTLs were above the significance level of –Log10 *P > *1.3 (**Table 3**). Checking the Q-Q plots for this trait (**Figure 1E**), indicated that the ANOVA, GLM, and SUPER models did not control false positives well in a diverse population. The FDR and PFDR methods gave more false positives than the Bonferroni method in the ANOVA, GLM, and SUPER models (**Table 3**). Complex models (MLM, CMLM, ECMLM, and MLMM), which were expected to control false positives arising from population structure and family relatedness, did not identify any significant associations after performing multiple comparisons adjustments. These complex models reduced the *P*-value inflations (**Table 3**), which led to an increase in the false negative error rates. For the FarmCPU model, the Bonferroni adjustment identified 4 out of 20 highly significant associations, which means that these methods gave 16 false negative associations; the false discovery rate and positive false discovery rate adjustments with the FarmCPU model, identified 10 out of 20 highly significant associations, resulting in 10 false negatives above the selected cutoff value of –Log10 *P >* 1.3 (which is an equivalent to the *P* < 0.05) after adjustments (**Table 3**). Without any multiple comparison adjustments, FarmCPU identified 19 out 20 associations at a cutoff of 3.5 (–Log10 (*P*) ≥ 3.5; *P* ≤ 0.0003).

## Discussion

AM is based on the LD of marker with a QTL and is a popular approach for fine mapping traits of interest. LD in an AM population can also result from population structure, family relatedness, selection, and genetic drift (Flint-Garcia et al., 2003; Yu et al., 2006), which are the major reasons of false positive associations. The success of AM to identify true associations depends on the ability to separate LD of the marker with a QTL from LD due to other causes. There is a need for an appropriate model that can correctly identify LD caused by population structure and family relatedness.

In this study, eight different statistical models, ranging from single to multilocus, were compared for AM of three empirical phenotype traits differing in heritability in two crop species, soybean and maize, that vary in LD decay rates. The power of SNP identification is determined by several factors including the size of the population, the population structure, the extent of LD in the population, the heritability and underlying genetic architecture of the trait (Yu et al., 2006). For all previously reported traits, several SNPs were identified in this study, which indicated that all these traits were complex, quantitative traits, controlled by a large number of genes with small effects. The power of detecting SNP and mapping resolution for complex traits depend on the LD exploited in the population by the statistical model (Yu et al., 2006). As expected, faster LD decay over physical distance was observed in maize compared to soybean because maize is a cross-pollinated with a higher recombination rate and soybean is a self-pollinated with a lower recombination rate.

Based on the Q-Q plots, we observed a nonuniform distribution of *P* values in the ANOVA, GLM, and SUPER models of all empirical traits (**Figures 1** and **2**). These results are similar to previous studies (Yu et al., 2006; Stich et al., 2008; Zhao et al., 2011) indicating that these models are inappropriate for AM of complex traits in plants because they generate spurious marker-trait associations. Complex models including MLM, CMLM, and ECMLM were proposed to correct population structure and family relatedness (Yu et al., 2006). We observed a straight line close to the 1:1 line with slightly deviated tail in the Q-Q plots of MLM, CMLM, and ECMLM, indicating that these models reduced the false positives, but increased false negatives because most significant markers were present close to the 1:1 line. These false negatives were generated due to the overfitting of these complex models. Similar results were observed in other studies (Wen et al., 2015; Tamba et al., 2017; Li et al., 2018; Wen et al., 2018) where these complex models generated more false negatives. In contrast, the Q-Q plot of the FarmCPU model, a multilocus model, controlled both false positives and false negatives as indicated by a straight line (close to the 1:1 line) with a sharp deviated tail for all empirical traits in both crops.

Some studies, where multilocus models, including mrMLM (Wang et al., 2016), FASTmrEMMA (Wen et al., 2018), and LASSO (ISIS EM-BLASSO) (Tamba et al., 2017), were used, performed better than MLM-based models. Liu et al. (2016) reported that the FarmCPU model avoids overfitting by using two types of adjustments for testing markers. The first type of adjustment was fitting covariates of population structure, family relatedness, and pseudo-quantitative trait nucleotides; the second type of adjustment either refines how family relatedness is derived from all the markers, or selectively includes or excludes pseudo-quantitative trait nucleotides based on their relationship with the testing markers.

These eight AM models were also compared based on simulated traits in which a known number of QTLs were simulated. Among these models, the FarmCPU model identified the number of QTLs close to the number of simulated QTLs for all traits in both crops. Comparison of Q-Q plots of different models for all simulated traits indicated that the FarmCPU controlled better the false positives and false negatives. Additionally, FarmCPU identified markers of qualitative traits closer to the published location of genes controlling these traits compared to the other models. Instead of providing a large peak as in other models, the FarmCPU model provided a single most significant marker, which was always present closest to the published genes.

For determining statistical significance in AM, different multiple comparison methods are used with a cutoff of *P* = 0.05, and several of these methods were compared when used in combination with the eight AM models. Complex models (MLM, CMLM, ECMLM, and MLMM) were particularly conservative and did not find any markers after adjustment; these complex models and multiple comparison methods are apparently increasing the number of false negatives. In contrast, ANOVA, GLM, and SUPER models identified more than 20 QTLs after multiple comparison adjustments, indicating that these models increased the false positives. In contrast, the FarmCPU model performed better than other models for these multicomparison adjustments by identifying 10 QTLs with less conservative methods, FDR and PFDR. Based on the Q-Q plots and the number of known simulated QTLs, the FarmCPU was an appropriate model for controlling false positives and false negatives compared to other models. Other multiple comparison methods were overly conservative for selection of significant threshold for AM. Determination of the correct significant threshold for AM can be determined by an empirical relationship based upon marker-based heritability (Kaler and Purcell, 2019).

## Conclusions

This study compared eight statistical models for AM of three empirical phenotypic traits differing in heritability and six simulated traits in two crop species, soybean, and maize, varying in LD decays rates. Based on the Q-Q plots and the number of known simulated QTLs, the FarmCPU was an appropriate model for controlling false positives and false negatives compared to other models. These finding were also supported by the AM of six qualitative traits, which identified a single most significant SNP closest to the known published genes. The FarmCPU model performed better for multiple comparison adjustments compared to other models because adjustments were overly conservative for MLM, CMLM, ECMLM, and MLMM and did not find any QTL. In contrast, for ANOVA, GLM, and SUPER models, these adjustments found more than 20 QTLs. From this study, we conclude that FarmCPU provides a robust model for AM of complex traits in plants, which effectively controls both false positives and false negatives.

## Data Availability Statement

All datasets generated for this study are included in the article/**Supplementary Material**.

## Author Contributions

AK and LP conceived of the idea and wrote the manuscript. AK performed simulations and data analysis. JG and TB provided theoretical insights and valuable edits. All authors read and approved the final version.

## Funding

Partial funding from this project was from the United Soybean Board (project #1920-172-0116-A). Additional funds were provided by the University of Arkansas System, Division of Agriculture and the USDA-ARS.

## Conflict of Interest

The authors declare that the research was conducted in the absence of any commercial or financial relationships that could be construed as a potential conflict of interest.
