# Supplementary material for: Comparing Different Statistical Models and Multiple Testing Corrections for Association Mapping in Soybean and Maize
Source: Front Plant Sci. 2020 Feb 25;10:1794. doi: 10.3389/fpls.2019.01794 (PMC7052329; doi:10.3389/fpls.2019.01794)
Supplement: Supplementary file 3 [file DataSheet_3.docx]

**Table S1.** Example of R script that was used to simulate a trait, having Heritability=60% and number of QTLs=20 (H60_Q20), using the marker data of 31,260 SNPs for 346 soybean accessions.

#Read Soybean Genotype data file

D<- read.big.matrix("GN.txt", type="char", sep="\t", head = TRUE)

dim(D)

D=D[,2:31261]

D1=as.data.frame(as.matrix(D))

t(D1)

D2=t(D1)

QTL <- 100*(1:20) #pick 20 QTL

u <- rep(0,31620) #marker effects

u[QTL] <- 1

g <- as.vector(crossprod(D2,u))

h2 <- 0.6 #heritability

y <- g + rnorm(346,mean=0,sd=sqrt((1-h2)/h2*var(g)))

#Saving simulated file

write.table(y, "H60Q20.txt", sep="\t")

**Table S2.** Marker distribution in Maize and Soybean.

| **Chromosome** | **Maize** |  | **Soybean** |
| --- | --- | --- | --- |
| 1 | 7,592 |  | 1,238 |
| 2 | 5,732 |  | 1,924 |
| 3 | 5,493 |  | 1,292 |
| 4 | 5,373 |  | 1,415 |
| 5 | 5,326 |  | 1,352 |
| 6 | 3,988 |  | 1,360 |
| 7 | 4,056 |  | 1,593 |
| 8 | 4,163 |  | 1,884 |
| 9 | 3,590 |  | 1,448 |
| 10 | 3,520 |  | 1,622 |
| 11 |  |  | 1,220 |
| 12 |  |  | 1,094 |
| 13 |  |  | 2,019 |
| 14 |  |  | 1,587 |
| 15 |  |  | 1,911 |
| 16 |  |  | 1,436 |
| 17 |  |  | 1,586 |
| 18 |  |  | 2,595 |
| 19 |  |  | 1,624 |
| 20 |  |  | 1,060 |
|  |  |  |  |
| Total Number | 48,833 |  | 31,260 |
