# Supplementary figures and images for: Comparing Different Statistical Models and Multiple Testing Corrections for Association Mapping in Soybean and Maize
Source: Front Plant Sci. 2020 Feb 25;10:1794. doi: 10.3389/fpls.2019.01794 (PMC7052329; doi:10.3389/fpls.2019.01794)

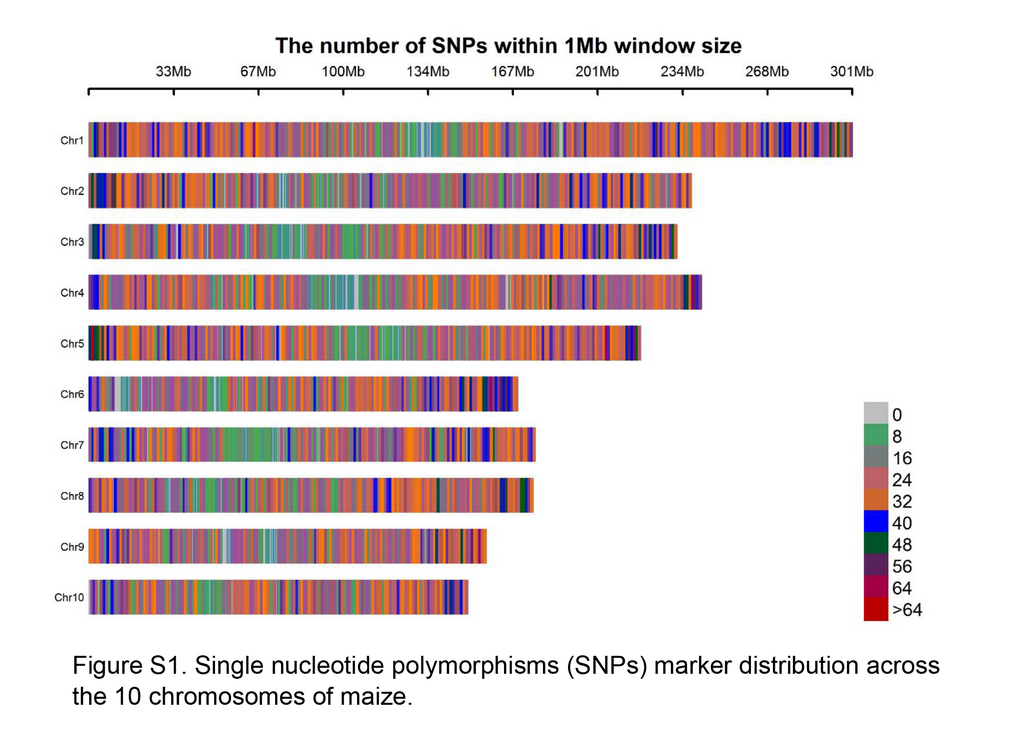

Supplement: Supplementary file 4 [file Image_1.tif]

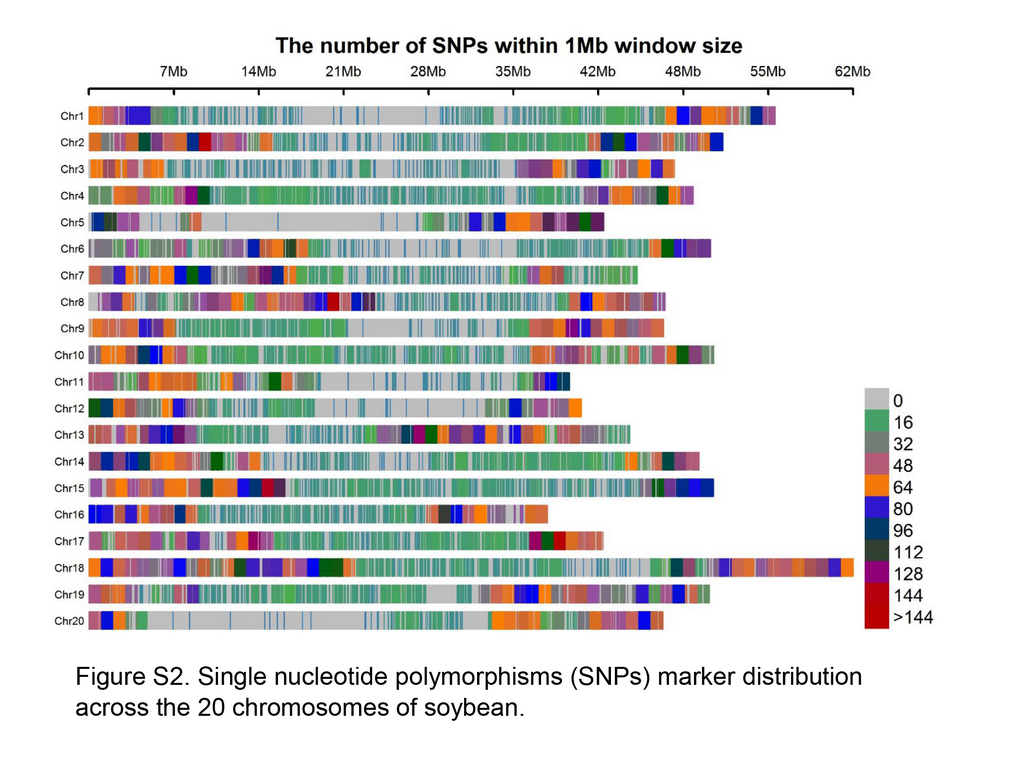

Supplement: Supplementary file 5 [file Image_2.tif]

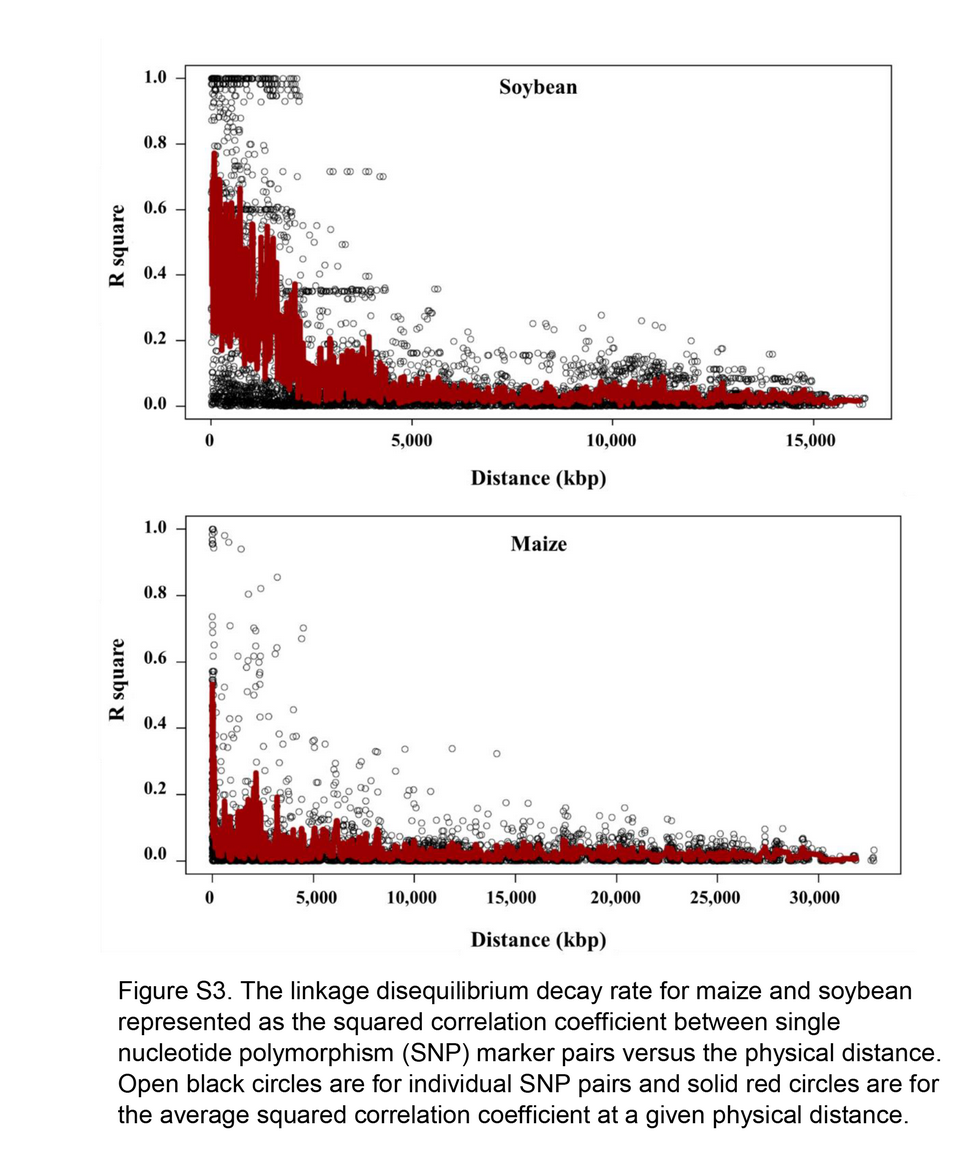

Supplement: Supplementary file 6 [file Image_3.tif]
